# Editorial: Balancing act: exploring the impact of steroid hormones, diets/supplements, and new drugs on renal function

**DOI:** 10.3389/fphys.2025.1768413

**Published:** 2026-01-07

**Authors:** Guiomar Nascimento Gomes, Ricardo Fernandez, Deise Carla Almeida Leite-Dellova, Dulce Elena Casarini, Licy L. Yanes Cardozo

**Affiliations:** 1 Department of Physiology, Federal University of São Paulo, São Paulo, Brazil; 2 Department of Physiology, Federal University of Paraná, Paraná, Brazil; 3 Department of Veterinary Medicine, Faculty of Animal Science and Food Engineering, University of São Paulo, Pirassununga, Brazil; 4 Department of Medicine, Federal University of São Paulo, São Paulo, Brazil; 5 Departments of Medicine (Endocrinology) and Pharmacology and Toxicology, University of Mississippi Medical Center, Jackson, MS, United States

**Keywords:** gender-affirming therapy, kidney disease, molecular signaling pathways, sex steroids, tubular cells prolifaration

Kidney disease (KD) is a major public health problem affecting millions of people worldwide. In addition to well-established factors such as hypertension and diabetes mellitus, sex/gender differences are increasingly recognized as an influencing factor in the development and progression of KD; however, the specific role of sex steroids in renal function and disease is not yet completely known. On the other hand, new classes of drugs with nephroprotective functions are being developed, especially for diabetic patients who have an increased risk of developing this disease.

In this special edition, we have gathered articles that address various aspects of renal pathophysiology, with a strong emphasis on personalized approaches to both risk assessment and treatment.

Sex steroids have been widely used as an essential tool in gender-affirming therapy (GAT). However, few studies have evaluated the impact of GAT on renal function. In the study by Lichtenecker et al., the role that sex hormones play in renal function when administered as GAT, mimicking the condition of transgender people, was studied. In this study, GAT impacts renal function differently in male and female rats. Females treated with testosterone showed increased expression of sodium transporters and reduced sodium excretion, which may predispose them to hypertension. On the other hand, in males, the combination of estradiol used decreased glomerular filtration rate, suggesting a maladaptive response. Considering that the incidence of chronic kidney disease is increasing, including in transgender patients (Collister et al., 2021), the Lichtenecker study provides valuable translational evidence, indicating that GAT can lead to adaptive functional changes that may affect the individual’s health, justifying renal monitoring in transgender individuals undergoing GAT.

Also, in an experimental model mimicking GAT used by transgender people, Almeida et al. present interesting results in a protocol in which female rats were treated with testosterone associated or not with a resistance exercise program. These authors confirmed that GAT in females induces changes in renal function. Still, resistance exercise attenuated these effects, reducing macrophage infiltration and cell proliferation and decreasing glomerular tubularization (the presence of cells within Bowman’s capsule with features of proximal convoluted tubule epithelial cells). This study suggests that exercise can help reduce renal changes associated with GAT.

Kidney stones, regarded as a painful and debilitating condition, are increasingly linked to metabolic health and obesity. The study by Ding et al. provides valuable insights into this relationship by analyzing data from the National Health and Nutrition Examination Survey (NHANES). The study analyzed NHANES 2007–2018 data to assess kidney stone prevalence across metabolic health and weight categories. Kidney stone history was self-reported, and the condition was most common among individuals with obesity, regardless of metabolic status. Both metabolically healthy obesity (MHO) and metabolically unhealthy obesity (MUO) were associated with significantly higher odds of kidney stones compared with normal-weight, metabolically healthy individuals. Higher physical activity appeared to reduce risk, especially among those with MUO. Overall, obesity itself emerged as a key factor linked to kidney stone history.

Expanding the discussion on metabolic influences in kidney-related disorders, the study by Chen and Yin investigates the relationship between blood urea nitrogen (BUN) and the risk of hyperuricemia (HUA) in U.S. adults, with particular attention to sex differences. Using NHANES data from 1999 to 2020, the authors report that higher BUN levels are associated with an increased risk of HUA in the general adult population; however, the association may be stronger in females than in males. This disparity may stem from physiological differences, including the influence of sex hormones on uric acid metabolism and variations in renal clearance. The study underscores the importance of considering sex-specific factors in the diagnosis and management of conditions such as gout and KD, supporting a more individualized approach to care. Given the growing prevalence of these disorders, the findings highlight the need for targeted strategies that address risk factors differently in men and women.

Turning to the molecular signaling pathways within the nephron, the experimental work by Nakamura et al. provides mechanistic insight into how hormonal pathways influence renal function. Their study explores the interaction between SGLT2 inhibitors and aldosterone/mineralocorticoid receptor (MR) signaling in the proximal tubule (PT), particularly in the context of diabetic kidney disease (DKD) and the hyperkalemia often induced by mineralocorticoid receptor blockers (MRBs). Experimental findings show that aldosterone enhances PT sodium transport (via sodium/hydrogen exchanger 3 and Na+/HCO3− cotransporter) and upregulates potassium channel genes (TWIK-1/Kcnk1 and TASK-2/Kcnk5), effects that are completely suppressed by esaxerenone (a relatively new MRB). In a diabetic rat model, esaxerenone improved kidney injury but led to hyperkalemia, whereas combining esaxerenone with dapagliflozin (a SGLT2 inhibitor) reduced hyperkalemia and further improved renal outcomes. Overall, the findings suggest that SGLT2 inhibitors may mitigate MRB-induced hyperkalemia and improve kidney outcomes.

Another good example of the importance of the proximal tubule in KD is the study of Lopes-Gonçalves et al. This study evaluates the effects of exogenous administration of apelin-13 in the renal ischemia/reperfusion (I/R) model, Apelin-13, an adipokine, is known to regulate glomerular hemodynamics and renal water balance. While recent studies show promising results for mitigating kidney injury, its specific tubular mechanisms and potential adverse effects remain undefined.Lopes-Gonçalves et al. found that initial administration of apelin-13 did not improve tubular injury (KIM-1 and NGAL), creatinine clearance, or plasma urea level after renal I/R. Furthermore, megalin downregulation by renal I/R was not prevented by apelin-13. Moreover, exogenous apelin-13 administration reduces tubular cell proliferation and impairs ERK1/2 phosphorylation after renal I/R. *In vitro* experiments have also shown that apelin-13 inhibits ERK1/2 phosphorylation and inhibits NHE3 activity in murine proximal tubular cells. These findings suggest that apelin-13 suppresses tubular proliferation and may impair the adaptive response to renal I/R injury.

Together, these six studies emphasize the multifaceted metabolic underpinnings of KD—from obesity and sex-specific metabolic variation to molecular signaling pathways within the nephron. They collectively highlight the importance of integrated, personalized approaches in understanding KD risk and improving therapeutic strategies.
